# Supplementary material for: Chromothripsis during telomere crisis is independent of NHEJ, and consistent with a replicative origin
Source: Genome Res. 2019 May;29(5):737–49. doi: 10.1101/gr.240705.118 (PMC6499312; doi:10.1101/gr.240705.118)
Supplement: Supplemental Material [file supp_gr.240705.118_Supplemental_file_1.zip › contigs/annotated_contigs/DB53/contig.2.DB53_length_266_mean_cov_2.25563909774.docx]

**DB53_length_266_mean_cov_2.25563909774**

```
CAATAACATGGCTGTATTGTTCCAAAGAAGG|AATATAGGTAAATA|TTCTTACAAAACACCAGGGGTCCAGTCTAGGTTCTGCTGCTC
 >chr7:154481639-154481670 - E=2e-07 p=4e-02  >chr8:130160886-130161107 - E=3e-122
ACTACACAGAAAGCCAATCACTGAGATGATGAGTATTGCCAGGAAAGAAGGCTTTCATCAGGTGTTGCAGGTGAGGCAATGGGAGATCA

GTCTCAAACCATCTCCCTGATTTACTAAAATTAGGGGTTTATATAGCAGGGAAAAAATGTAACTATGTGTGGGAAAATAGGAAGCAGGG

A
```
